# Supplementary material for: Novel Allele Detection Tool Benchmark and Application With Antibody Repertoire Sequencing Dataset
Source: Front Immunol. 2021 Oct 26;12:739179. doi: 10.3389/fimmu.2021.739179 (PMC8576399; doi:10.3389/fimmu.2021.739179)
Supplement: Supplementary file 1 [file Image_1.pdf]

Supplementary Figure 1

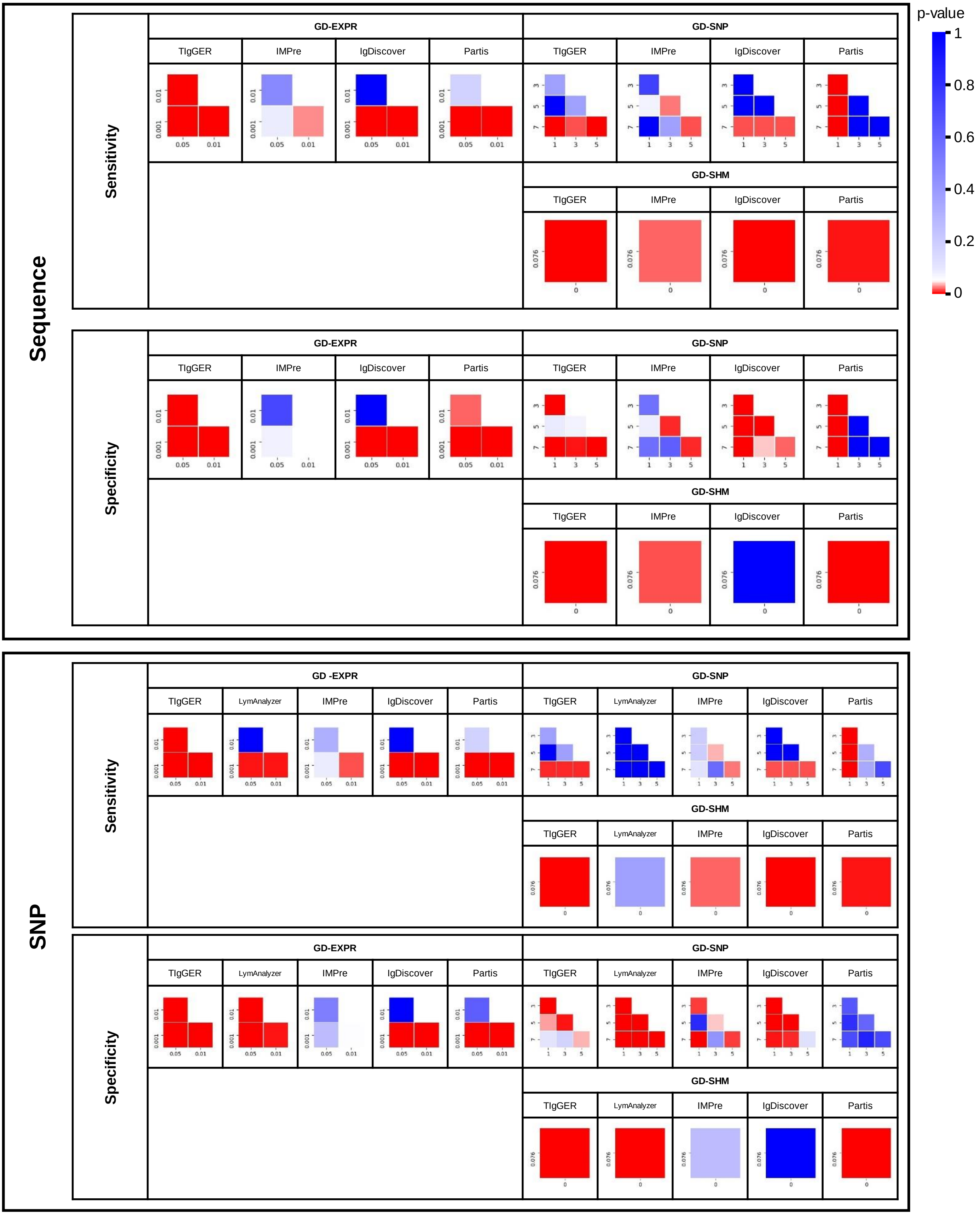

Supplementary Figure 1. Heatmap of double-tailed p-value of paired t-test between different subgroups with regard to sensitivity and specificity for different tools in genuine dataset. Each row or each column in a heatmap represents a subgroup. P-values below 0.05 are shown in red blocks and p-values above 0.05 are in blue blocks.

Supplementary Figure 2

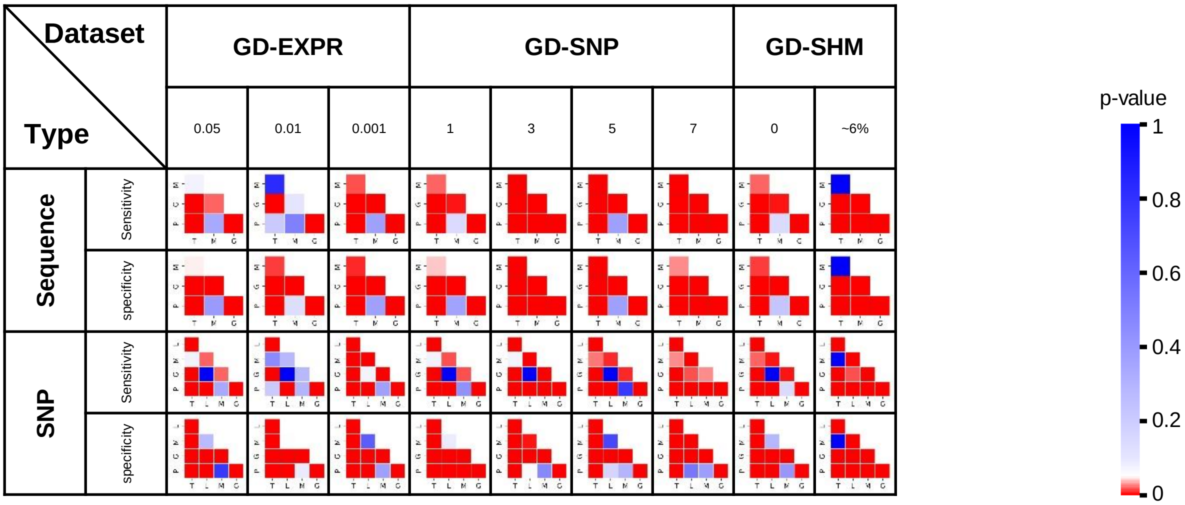

Supplementary Figure 2. Heatmap of double-tailed p-value of paired t-test between different tools with regard to sensitivity and specificity for different subgroups in real-world datasets. Each row or each column in a heatmap represents a unique tool (T, TIgGER; L, LymAnalyzer; M, IMPre; G, IgDiscover; P, Partis). P-values below 0.05 are shown in red blocks and p-values above 0.05 are in blue blocks.

Supplementary Figure 3

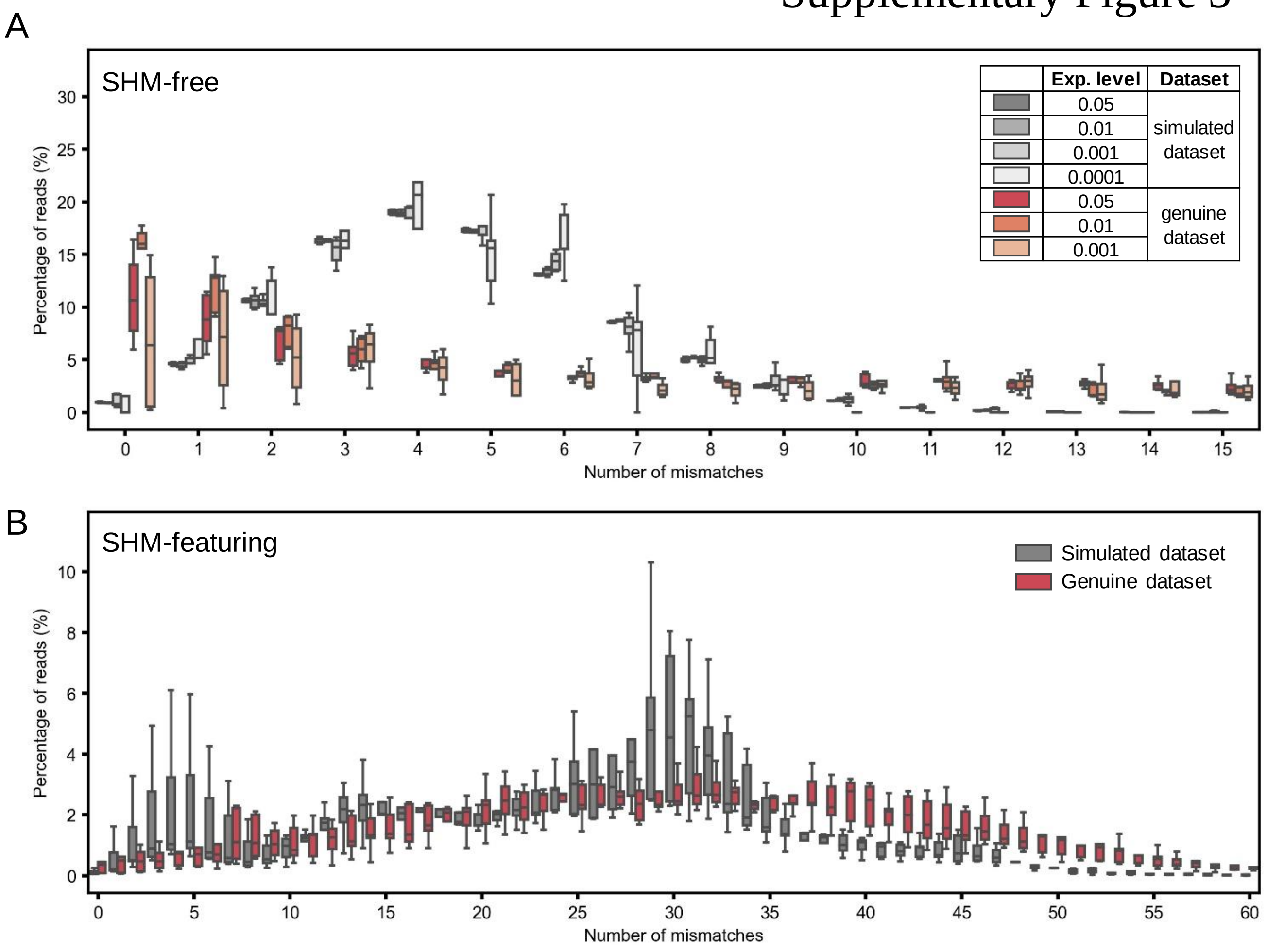

Supplementary Figure 3. Distribution comparisons of number of mismatches with germline reference for sequences from both simulated and genuine Ig-seq dataset. (A) Distribution comparison between DEXPR and GD-EXPR (SHM-free). **(B)** Distribution comparison between DSHM and GD-SHM (SHM-featuring). The mismatches are calculated based on the sequence annotation file used as TIgGER's input (see Materials and Methods).

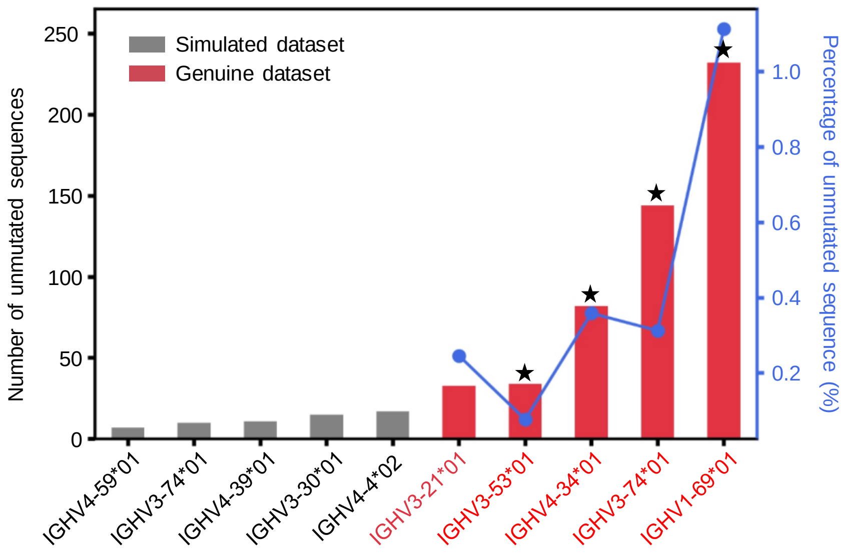

Supplementary Figure 4. Number and percentage of unmutated sequences for 10 novel alleles from simulated (DSHM) and genuine Ig-seq dataset (GD-SHM). Alleles marked with pentagrams were successfully discovered by *IgDiscover*. The "unmutated" sequences here represent those sequences identical to novel germlines after 8 nucleotides at the 3' end were removed, which is consistent with *IgDiscover*'s algorithm according to its documentation. The percentage of unmutated sequences is calculated as the number of unmutated sequences for an allele divided by the number of all sequences assigned to the same allele. This result indicates that a substantial amount of unmutated sequences are needed for *IgDiscover* when making successful discoveries.

Supplementary Figure 5

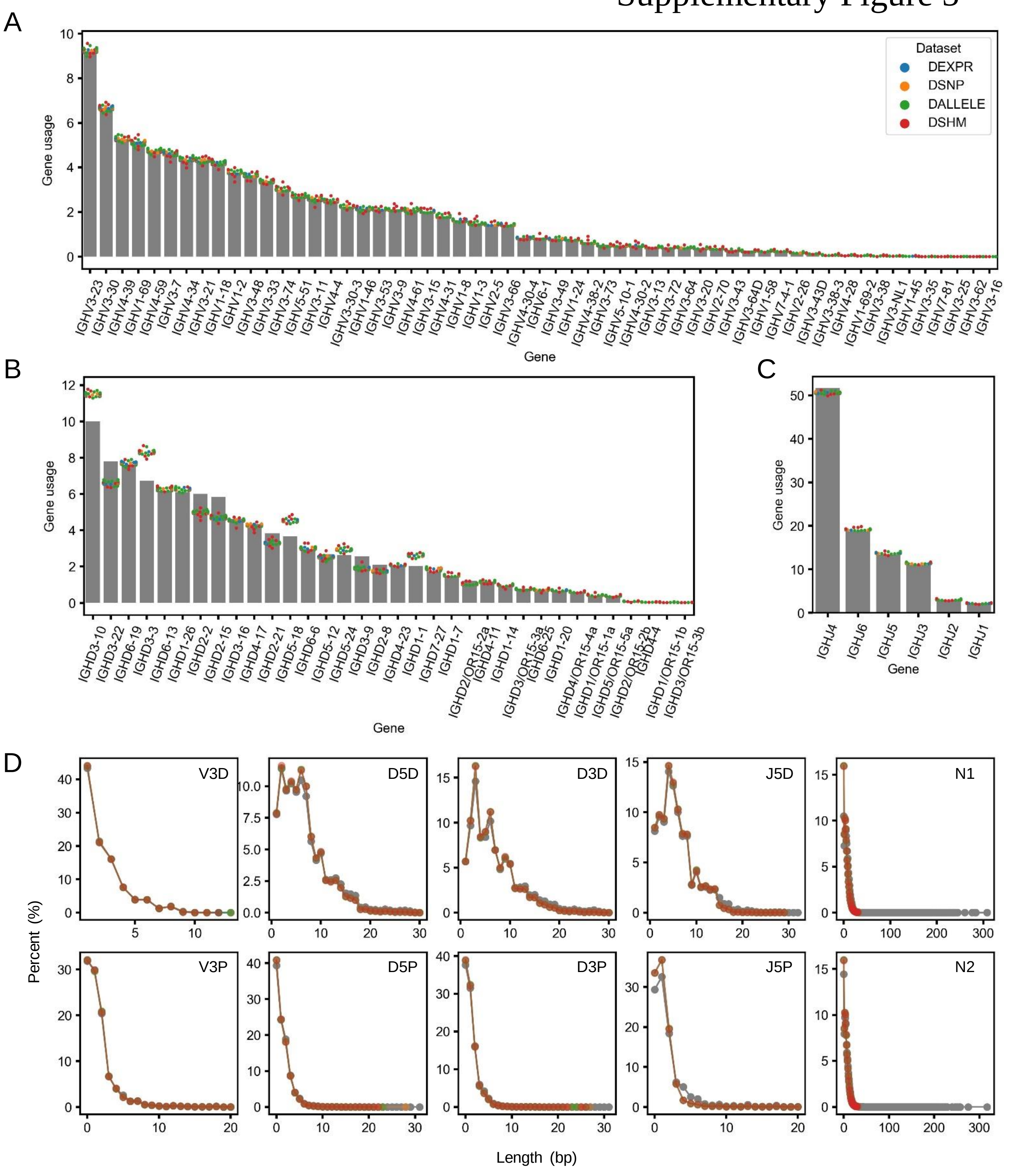

Supplementary Figure 5. Approximation of the real-world repertoire for repertoires in the four simulated datasets in this study. (A, B and C) Comparison of V, D and J gene usage between simulated datasets and real-world repertoires. Each dot overhanging a bar represents a simulated repertoire. **(D)** Comparison of the length distribution for 10 kinds of junction modification (i.e. V3D, D5D, D3D, J5D, V3P, D5P, D3P, J5P, N1 and N2; D, deleted nucleotide; P, palindromic nucleotide; N, nontemplated nucleotide) between simulated datasets and real-world repertoires. Mean values of all repertoires in a dataset were shown in (D). Note that gray bars in (A, B and C) and gray dots in (D) represent parameters obtained from real-world repertoires.
