# Supplementary material for: Novel Allele Detection Tool Benchmark and Application With Antibody Repertoire Sequencing Dataset
Source: Front Immunol. 2021 Oct 26;12:739179. doi: 10.3389/fimmu.2021.739179 (PMC8576399; doi:10.3389/fimmu.2021.739179)
Supplement: Supplementary file 2 [file Table_1.docx]

| **Supplementary Table 1.** Score of 10 key features of 5 NADTs | | | | | | | | | | | |
| --- | --- | --- | --- | --- | --- | --- | --- | --- | --- | --- | --- |
| **Tool** | **Supported receptor type(s)** | **Supported chain(s)** | **Supported gene(s)** | **Nonhuman species supported** | **Ease of installation** | **Ease of usage** | **Accepted sequence format** | **Parameter customizability** | **INDEL detection supported** | **Detail degree of output** | **Total score** |
| TIgGER | BCR (8) | IGH, IGK, IGL (8) | V (8) | Yes (10) | Easy (10) | Easy (10) | Annotated sequence (5) | High (10) | No (0) | High (10) | **79/100** |
| IMPre | BCR, TCR (10) | IGH, IGK, IGL, TRB, TRA (10) | V, J (9) | Yes (10) | Easy (10) | Easy (10) | FASTA (8) | High (10) | Yes (10) | Middle (8) | **95/100** |
| IgDiscover | BCR (8) | IGH, IGK, IGL (8) | V, D, J (10) | Yes (10) | Relatively easy (5) | Easy (10) | FASTA/SE FASTQ/PE FASTQ (10) | High (10) | Yes (10) | High (10) | **91/100** |
| LymAnalyzer | BCR, TCR (10) | IGH, IGK, IGL, TRB, TRA (10) | V, J (9) | Yes (10) | Easy (10) | Easy (10) | SE FASTQ (8) | No (0) | No (0) | Low (5) | **72/100** |
| Partis | BCR (8) | IGH, IGK, IGL (8) | V (8) | Yes (10) | Relatively easy (5) | Easy (10) | FASTA/SE FASTQ/CSV/YAML (10) | High (10) | No (0) | High (10) | **79/100** |
| Note, all features here were scored in a subjective manner. Numbers in parentheses indicate the scores for compared features. | | | | | | |  |  |  |  |  |
