# Supplementary material for: Novel Allele Detection Tool Benchmark and Application With Antibody Repertoire Sequencing Dataset
Source: Front Immunol. 2021 Oct 26;12:739179. doi: 10.3389/fimmu.2021.739179 (PMC8576399; doi:10.3389/fimmu.2021.739179)
Supplement: Supplementary file 3 [file Table_2.docx]

| **Supplementary Table 2.** Two unique novel germline sequences identified based on single naïve B cell sequencing dataset | | | | | | |
| --- | --- | --- | --- | --- | --- | --- |
| **Nearest known allele** | **# Supportive contigs^a^** | **Length (bp)** | **Start** | **End** | **SNP loci^b^** | **Individual** |
| IGHV5-51*01 | 35 (224, 0.16) | 295 | 1 | 295 | G3A, G13A, G23T, A29G, G57A, A67C, G70A, G71C, G83C, C84A, T87C, A88T, C93T, C96T, T119C, A128G, G135T, G138A, A142G, G147A, C153G, T159C, T162G, T168A, C173T, G176A, A177G, C183T, C189T, A200G, G201T, A213G, T227C, A229T, G230C, C231T, G235A, A259C, A260G, G262A, G279C, T288C | Donor1 |
| IGHV1-NL1*01 | 15 | 296 | 1 | 296 | T26C, C27T, C28G, G44C, T67C, G104A, T112C, A117G, A118G, N136-, A142T, A150C, C165T, C188A, T193C, A226G, N253A, C264T, A284C |  |
| IGHV1-NL1*01 | 16 | 296 | 1 | 296 | T26C, C27T, C28G, G44C, T67C, G104A, T112C, A117G, A118G, N136-, A142T, A150C, C165T, C188A, T193C, A226G, N253A, C264T, A284C | Donor2 |
| **Note:** a. Numbers in parentheses denote the number of contigs supportive of its known germline variant and the ratio of the two germline variants.  b. The indexes in SNP loci are 1-based. | | | | | | |
