# Supplementary material for: Novel Allele Detection Tool Benchmark and Application With Antibody Repertoire Sequencing Dataset
Source: Front Immunol. 2021 Oct 26;12:739179. doi: 10.3389/fimmu.2021.739179 (PMC8576399; doi:10.3389/fimmu.2021.739179)
Supplement: Supplementary file 5 [file Table_4.pdf]

Supplementary Table 4. Detailed information about the 24 unique NACs identified from 424 Ig-seq datasets amplified using RACE protocol

Index	Allele	SNP loci	SNP type	# Samples	# Donors	Isotypes	Tools				Source													
							TIgGER	*IMPre*	*IgDiscover*	*Partis*	*IMGT*	*VBASE2*	*IgPdb*	*Lym1K*	*OGRDB*	*Bernat_et_al*	*Thornqvist_et_al*	*Wang_et_al*	*Wendel_et_al*	*Corcoran_et_al*	*Gidoni_et_al*	*Mikocziova_et_al*	*Gadala-Maria_et_al_2019*	*Gadala-Maria_et_al_2015*
1	IGHV1-69*08	C170T	R	29	29	M	√	√	√				√											
2	IGHV4-38-2*02	A67G	R	16	16	M	√	√	√				√											
3	IGHV5-51*01	C42G	S	12	12	M	√	√	√				√	√										
4	IGHV3-7*03	G129A	S	8	8	M	√		√		√			√								√		
5	IGHV3-64D*06	G234T	S	7	7	M	√		√	√	√										√	√		
6	IGHV3-64*05	G241C	R	7	7	M	√		√		√		√									√		
7	IGHV3-13*01	G263A,T273C	R, S	7	7	M	√	√	√					√								√		
8	IGHV4-61*02	A213G	R	6	6	M	√	√	√													√	√	
9	IGHV3-33*01	G72C	S	3	3	M, G	√	√	√	√			√	√								√		
10	IGHV1-3*01	T32A	R	2	2	M	√		√		√			√								√		
11	IGHV4-39*07	C267A	S	2	2	M	√	√	√	√												√	√	
12	IGHV1-69*04	C163T	R	1	1	M	√	√	√				√									√		
13	IGHV4-39*07	A30G,T55A,C105T,A161T,A197G,G217A,C288T	S, R, S, R, R, R, S	1	1	G			√	√														
14	IGHV3-21*01	A163G,T169A,A170C	R, R, R	1	1	M	√	√	√	√														
15	IGHV3-9*01	T283C	R	1	1	M	√	√														√		
16	IGHV4-39*01	C63G	S	1	1	M	√	√						√								√		
17	IGHV4-30-2*01	G67A	R	1	1	M	√	√	√													√		
18	IGHV4-61*01	A38G	R	1	1	M	√		√													√		
19	IGHV1-69*01	C219T	S	1	1	M	√		√		√											√		
20	IGHV3-66*02	G276A	S	1	1	M	√		√															
21	IGHV4-59*01	T73C	R	1	1	M	√			√														
22	IGHV3-43D*04	G4A	R	1	1	M	√		√															
23	IGHV2-70*04	A14G	R	1	1	M	√		√															
24	IGHV4-39*01	G294A	S	1	1	M		√	√															

Note: The index in SNP loci column is 1-based. In the SNP type column, R denotes replacement SNP while S denotes silent SNP. Their order match the SNPs in SNP loci column. The ticks present in Tools and Source columns indicate the successful identification of NACs for a certain tool or inclusion of NACs in a typical source. The isotypes column indicates the type of dataset from which a NAC was identified (M, IgM; G, IgG). NACs highlighted in red are not included in the collected novel germline sequences.
