# Supplementary material for: Novel Allele Detection Tool Benchmark and Application With Antibody Repertoire Sequencing Dataset
Source: Front Immunol. 2021 Oct 26;12:739179. doi: 10.3389/fimmu.2021.739179 (PMC8576399; doi:10.3389/fimmu.2021.739179)
Supplement: Supplementary file 6 [file Table_5.pdf]

Supplementary Table 5. Detailed information about the 22 unique NACs identified from 263 Ig-seq datasets amplified using multiplex protocol

							Tools				Source													
Index	Nearest allele	SNP loci	SNP type	# Samples	# Donors	Isotype	*TIgGER*	*IMPre*	*IgDiscover*	*Partis*	*Bernat_et_al*	*Corcoran_et_al*	*Gadala-Maria_et_al_2015*	*Gadala-Maria_et_al_2019*	*Gidoni_et_al*	*IMGT*	*IgPdb*	*Lym1K*	*OGRDB*	*Mikocziova_et_al*	*Thornqvist_et_al*	*VBASE2*	*Wang_et_al*	*Wendel_et_al*
1	***IGHV1-69*04***	G97A	R	9	6	M	√	√									√							
2	IGHV1-18*01	T78A, T79G, A80G, T87C, C89G, G98C	S, R, R, S, R, R	4	3	M	√	√																
3	IGHV4-30-4*02	A267C	S	3	3	M	√	√				√				√		√						
4	***IGHV4-39*07***	C267A	S	3	2	M	√	√						√						√				
5	IGHV1-69*13	C170T	R	3	1	M	√	√		√							√							
6	***IGHV4-61*02***	A213G	R	3	1	M	√	√		√				√			√			√				
7	IGHV1-69*13	G220A	R	2	2	M	√	√				√				√	√	√						
8	IGHV3-21*01	A97C	R	2	2	M	√			√														
9	IGHV4-34*01	A205G	R	2	1	M	√	√										√						
10	IGHV1-69*10	C163T	R	1	1	M	√	√					√	√		√	√							
11	IGHV1-69*13	A78T, G79T, G80A, C87T, G89C, C98G	S, R, R, S, R, R	1	1	M	√	√																
12	IGHV1-8*01	A80C	R	1	1	M	√			√														
13	IGHV3-13*01	G197A	R	1	1	M	√			√								√						
14	IGHV3-30*02	T180C	S	1	1	M	√	√									√							√
15	IGHV3-74*01	A117C	R	1	1	M	√			√														
16	IGHV3-9*01	A117C	R	1	1	M	√	√																
17	IGHV4-30-4*02	A98G, A267C	R, S	1	1	M	√	√												√				
18	IGHV5-51*01	C84T, A88G, C89T, G92C, C96T, G138A, G169A, G279T	S, R, R, R, S, S, R, R	1	1	G			√	√														
19	IGHV5-51*01	G104C, G147A, G279A	R, S, R	1	1	M		√	√															
20	IGHV5-51*01	G251C	R	1	1	M		√	√															
21	IGHV5-51*01	G92A, C153T, C183T, G230A	R, S, S, R	1	1	M		√	√															
22	IGHV6-1*01	A94C	R	1	1	M	√	√																

Note: The index in SNP loci column is 1-based. In the SNP type column, R denotes replacement SNP while S denotes silent SNP. Their order match the SNPs in SNP loci column. The ticks present in Tools and Source columns indicate the successful identification of NACs for a certain tool or inclusion of NACs in a typical source. Isotypes column indicates the type of dataset, from which a NAC was identified (M, IgM; G, IgG). NACs highlighted in red are not included in the collected novel germline sequences. The sequences of NACs in bold and italic are included in those identified from RACE dataset (Supplementary Table 4).
